# Cardiac Mitochondrial Respiratory Dysfunction and Tissue Damage in Chronic Hyperglycemia Correlate with Reduced Aldehyde Dehydrogenase-2 Activity

**DOI:** 10.1371/journal.pone.0163158

**Published:** 2016-10-13

**Authors:** Vishal R. Mali, Guodong Pan, Mandar Deshpande, Rajarajan A. Thandavarayan, Jiang Xu, Xiao-Ping Yang, Suresh S. Palaniyandi

**Affiliations:** 1 Division of Hypertension and Vascular Research, Department of Internal Medicine, Henry Ford Health System, Detroit, MI, United States of America; 2 Department of Cardiovascular Sciences, Center for Cardiovascular Regeneration, Houston Methodist Research Institute, Houston, TX, United States of America; 3 Department of Physiology, Wayne State University, Detroit, MI, United States of America; Temple University, UNITED STATES

## Abstract

Aldehyde dehydrogenase (ALDH) 2 is a mitochondrial isozyme of the heart involved in the metabolism of toxic aldehydes produced from oxidative stress. We hypothesized that hyperglycemia-mediated decrease in ALDH2 activity may impair mitochondrial respiration and ultimately result in cardiac damage. A single dose (65 mg/kg; i.p.) streptozotocin injection to rats resulted in hyperglycemia with blood glucose levels of 443 ± 9 mg/dl *versus* 121 ± 7 mg/dl in control animals, p<0.0001, N = 7–11. After 6 months of diabetes mellitus (DM) induction, the rats were sacrificed after recording the functionality of their hearts. Increase in the cardiomyocyte cross sectional area (446 ± 32 μm^2^ *Vs* 221 ± 10 μm^2^; p<0.0001) indicated cardiac hypertrophy in DM rats. Both diastolic and systolic dysfunctions were observed with DM rats compared to controls. Most importantly, myocardial ALDH2 activity and levels were reduced, and immunostaining for 4HNE protein adducts was increased in DM hearts compared to controls. The mitochondrial oxygen consumption rate (OCR), an index of mitochondrial respiration, was decreased in mitochondria isolated from DM hearts compared to controls (p<0.0001). Furthermore, the rate of mitochondrial respiration and the increase in carbonyl cyanide-p-trifluoromethoxyphenylhydrazone (FCCP)-induced maximal respiration were also decreased with chronic hyperglycemia. Chronic hyperglycemia reduced mitochondrial OXPHOS proteins. Reduced ALDH2 activity was correlated with mitochondrial dysfunction, pathological remodeling and cardiac dysfunction, respectively. Our results suggest that chronic hyperglycemia reduces ALDH2 activity, leading to mitochondrial respiratory dysfunction and consequently cardiac damage and dysfunction.

## Introduction

Hyperglycemia, the increase in blood glucose levels, is a common symptom of both type-1 and type-2 diabetes mellitus (DM). Though the etiopathogenesis is different for both types [1–4], proper control of blood glucose levels is necessary to avoid severity of diabetic complications in both types of diabetic patients [1, 5–8]. The morbidity and mortality of diabetic patients is associated with cardiovascular complications, with percentages as high as 44% [9] and 65% [10] in type-1 and type-2 diabetes, respectively. Chronic hyperglycemia-induced oxidative stress is considered to be one of the major causes of diabetic cardiovascular complications [11, 12]. Therefore, understanding the molecular pathogenesis of hyperglycemia-induced oxidative stress in the myocardium would be beneficial for diabetics in general.

Hyperglycemia-induced overproduction of reactive oxygen species (ROS) by the mitochondrial electron-transport chain is a key aspect of the pathogenesis of diabetic cardiac damage [13]. Oxidative stress-induced ROS increases the generation of toxic reactive aldehydes such as 4-hydroxy-2-nonenal (4HNE) in the mitochondria [14]. Additional 4HNE then forms adducts with proteins, leading to cardiac damage [15, 16]. 4HNE is metabolized by aldehyde dehydrogenases (ALDHs) into 4-hydroxy-2-nonenoic acid (4HNA), a non-toxic acid [17]. ALDH2, the mitochondrial isoform of ALDH, plays an important role in detoxifying 4HNE, further protecting the heart from oxidative stress/injury [18, 19]. Reduced activity and levels of myocardial ALDH2 were reported in type-1 diabetic hearts [20, 21]. Recently, we found that 4HNE forms adducts with ALDH2 itself and attenuates its activity, thereby contributing to cardiac hypertrophy in a non-genetic model of type-2 DM [15]. Overexpression of ALDH2 conferred cardio protection in STZ-injected diabetic mice [20]. ALDH2 overexpression attenuated the decrease or drop in mitochondrial membrane potential [20]. ALDH2 activation by a small molecule activator, Alda-1, protected ischemia-reperfusion induced myocardial injury. Particularly, this compound was known to attenuate 4HNE-induced ALDH2 impairment [19]. In summary, ALDH2 activity seems to be vital in preventing 4-HNE-induced cardio toxicity irrespective of origin of the diseases.

Earlier it was reported that 4HNE adduct formation inhibits activity of cytochrome c oxidase (COX IV), a mitochondrial respiratory complex IV protein, and leads to myocardial injury with ischemia-reperfusion [16]. Similarly, 4HNE adduct formation with succinyl dehydrogenase (SDH) in the STZ-induced type-1 diabetic heart leads to inhibition of mitochondrial respiratory complex II activity [22]. In another study, 4HNE treatment attenuated mitochondrial respiration in cultured neonatal cardiomyocytes. Exhaustion of mitochondrial respiratory reserve capacity in cells puts them at risk of succumbing to oxidative stress [23]. All these studies demonstrate that 4HNE plays an important role in mitochondrial respiratory dysfunction in cardiac pathologies.

Based on these reported findings, we hypothesized that enhanced 4HNE levels and reduced ALDH2 activity lead to mitochondrial respiratory dysfunction and ultimately cardiac damage and dysfunction in DM. To test this hypothesis, we employed a rat model of streptozotocin (STZ)-induced chronic hyperglycemia. This model presents hyperglycemia, myocardial 4HNE accumulation, and cardiac damage; therefore it is suitable to test our hypothesis. Moreover this model is simple, easily reproducible and most importantly, it produces hyperglycemia as early as 2 days and remains elevated until several months, thus mimicking chronic hyperglycemia in type-1 diabetic patients without other co-morbid factors for cardiac diseases.

## Materials and Methods

### Induction of hyperglycemia

Hyperglycemia was induced in 8-week-old male Sprague-Dawley rats by administering a single intraperitoneal (i.p.) injection of streptozotocin (STZ) (65 mg/kg). It was prepared freshly in citrate buffer (pH 4.5) for maximal stability. The control group was injected with the vehicle only. To ensure that the animals were diabetic, after 48 hours of STZ injection, rats were fasted for 6 hours and their blood sample was collected from their tail veins and their glucose levels were measured with a glucometer. Rats with blood glucose values of >250 mg/dL 48 hrs after STZ injection were considered as diabetic and included in the study.

The animal protocol has been approved by the Henry Ford Health System Institutional Animal Care and Use Committee. It adheres to the guiding principles of the care and use of experimental animals in accordance with the NIH guidelines. Henry Ford Hospital operates on an AAALAC certified animal facility with licensed veterinarian and well-trained veterinary technicians. The rats were housed in our animal facility and provided with normal chow and 24 hour water access. On the day of STZ injection, the rats were provided with sucrose water to avoid hypoglycemia. Since diabetic animals urinate enormously, the bedding was changed frequently than control rats. The rats were housed in a separate and designated-restricted room immediately after STZ until they excrete urine completely and later moved to normal rooms.

Six months after DM induction, we assessed cardiac function by hemodynamic measurements.

At the end of the experiments, rats were anesthetized with sodium pentobarbital (50 mg/kg, i.p.), the chest opened and heart excised. The hearts were weighed, and stored appropriately at -80°C. A portion of fresh heart tissue was used to isolate mitochondria. The middle portions of the cardiac tissue were fixed with 10% formalin in PBS, embedded in paraffin as blocks, and several transverse sections were cut for histopathological studies.

### Mitochondrial isolation and measurement of oxygen consumption rate (OCR) in the isolated rat heart mitochondria

#### Reagent and solution preparation

Mitochondria isolation buffer (IBc): 10 ml of 0.1 M Tris–MOPS and 1 ml (0.1 M) of EGTA/Tris to 20 ml of 1M sucrose. The pH was adjusted to 7.4 and the volume was made to 100 ml with distilled water.

#### Components / formulation of mitochondrial assay solution-1 (MAS)

Sucrose 70 mM, mannitol 220 mM, KH2PO4 5mM, Mgcl2 5mM, HEPES 2 mM, EGTA 100 mM, fat free BSA 2%. MAS was prepared for the dilution of substrates, ADP and respiration reagents. Stocks of succinate (0.5 M) and ADP (0.5 M) were made in H2O and adjusted to pH 7.2 with potassium hydroxide. Stocks of 2.5 mM FCCP [carbonyl cyanide 4-(trifluoromethoxy)phenylhydrazone], 2.5 mM rotenone, 2.5 mM oligomycin and 2.5 mM antimycin A were made in DMSO and stored at -20°C.

#### CMST (Cell Mito Stress Test) media for cell bioenergetic measurements

1% glucose along with 1 mM sodium pyruvate and 2 mM GlutaMAX were added to the XF medium (Seahorse Bioscience).

#### Isolation of rat heart mitochondria

After hemodynamic measurements, approximately 400 mg of heart tissue was harvested and homogenized in mitochondrial buffer (IBc). This homogenate was centrifuged at 2000 RPM for 10 min at 4°C and the supernatant was collected and again centrifuged at 5000 RPM for 10 min at 4°C. The sedimented mitochondrial pellet was re-suspended in 50 μl of mitochondrial buffer. Mitochondrial protein was measured by means of Bradford assay and 4 μg of mitochondrial protein was added to each well of a collagen-coated plate. The plate was transferred to a centrifuge equipped with a swinging bucket microplate adaptor and spun at 2000 g for 20 minutes at 4°C. ADP, Oligomycin, FCCP and Antimycin A were loaded sequentially through ports in the Seahorse XF^e^96 FluxPak cartridge. The cartridge and the mitochondria coated plate were then transferred to the XF^e^96 Extracellular Flux Analyzer (Seahorse Bioscience) and the experiment was initiated.

### ALDH activity assay

ALDH2 activity was measured by the procedure described elsewhere [15, 19]. In brief, enzymatic activity of ALDH2 from cardiac tissue homogenate was determined spectrophotometrically by the reductive reaction of NAD+ to NADH at λ340 nm. All assays were carried out at 25°C in 0.1M sodium pyrophosphate buffer, *p*H = 9.5 with 2.4 mM NAD+ as a cofactor and 10 mM acetaldehyde as the substrate.

### Western blotting of 4HNE protein adducts and mitochondrial OXPHOS proteins

The Western blot was performed as described earlier [24, 25]. In brief, protein samples from cardiac homogenate were separated on SDS-polyacrylamide gels by electrophoresis and the proteins were then transferred to immobilon-P membranes (Millipore, Billerica, MA). Levels of 4HNE-protein adducts in heart samples were determined using antibodies of anti-4HNE-Cys/His/Lys rabbit antibody (Millipore) (at a concentration of 1:1000) and Total OXPHOS rodent WB Antibody Cocktail (Abcam) at a concentration of 1:15000. Porin mouse monoclonal antibody at a concentration of 1:2000 (Abcam) was used as a housekeeping marker for comparison. The bound antibody was visualized with horseradish peroxidase (HRP)-coupled, secondary antibody, and chemiluminescence detection reagents.

### Co-immunoprecipitation of ALDH2 with phospho antibodies

Co-immunoprecipitation (IP) studies were performed as we described earlier [26]. An anti-pSer/Thr (phe) antibody (CST Inc) was crosslinked to dimethyl pimelimidate as per Abcam Inc protocol. The cross linked antibody was used in normal co-IP protocol. Briefly, we used cardiac tissue protein (500ug) in a final volume of 200 μL and incubated it for 2 hours. Then protein-A/G agarose beads (Santa Cruz) were added to each sample and rocked at 4°C overnight. The beads were washed several times and then re-suspended in IP buffer. The samples were immunoblotted against the anti-ALDH2 antibody.

### Measurement of cardiomyocyte hypertrophy

Myocardial sections were stained with hematoxylin-eosin staining to measure cardiomyocyte hypertrophy. The details were mentioned in our previous study [15]. In brief, cardiomyocytes with a relatively circular shape and a centered nucleus were included for quantification of each high power field. The cross sectional area was measured for these cardiomyocytes. We scored at least 15 photomicrographs for each sample.

### Measurement of cardiac fibrosis

The myocardial sections were stained with Picrosirius red. The red color indicates the deposition of collagen and this area was measured using the MicroSuite software (Olympus America). The percent (%) area of fibrosis was quantified from each tissue section as previously described [27].

### Cardiac functional assessment by echocardiography

After 6 months of induction of the diabetes, the left ventricular dimension and function were assessed in anesthetized rats (1–2% isoflurane) using an echocardiograph equipped with a 15-MHz linear transducer (Acuson c256), as described previously [15].

### Cardiac function assessment by hemodynamic measurements

Cardiac function was assessed by using the Millar Mikro-Tip Pressure Catheter, SPR-320 (ADInstrumnts, Australia) as reported earlier by our lab [15]. In brief, the Millar catheter was inserted into the left ventricle of the rat *via* the right carotid artery to assess left ventricular pressure (LVP) and the peak and minimum values of LV dP/dt (LV dP/dtmax and LV dP/dtmin, respectively) under the influence of an anesthetic, Inactin (100 mg/kg i.p.). Hemodynamic parameters were recorded after stabilization for 30 min, using an eight-channel lab recorder (ADInstruments, Australia) containing LABCHART-7 software.

### Correlation analysis

A linear regression analysis between ALDH2 activity and mitochondrial function, ALDH2 activity and cardiac hypertrophy and fibrosis, and ALDH2 activity and systolic and diastolic dysfunction was performed. Individual correlation graphs were then plotted.

### Statistical analysis

Data are presented as mean ± standard error of the mean (SEM). To confirm the data is normally distributed, we used a non-parametric Mann-Whitney test in addition to Student t test using Graphpad Prism 5. Statistical significance was achieved when p was <0.05.

## Results

### Streptozotocin-induced chronic hyperglycemia decreased ALDH2 activity and levels and showed an increase in 4HNE protein adducts in rat hearts

A single dose (65 mg/kg; i.p.) injection of STZ resulted in hyperglycemia, as evident from increased blood glucose levels {443 ± 9 mg/dl in DM *versus* 121 ± 7 mg/dl in control animals} within a week, which persisted for 6 months. The chronic hyperglycemia resulted in a 50% decrease in body weight relative to control rats.

**Fig 1:**
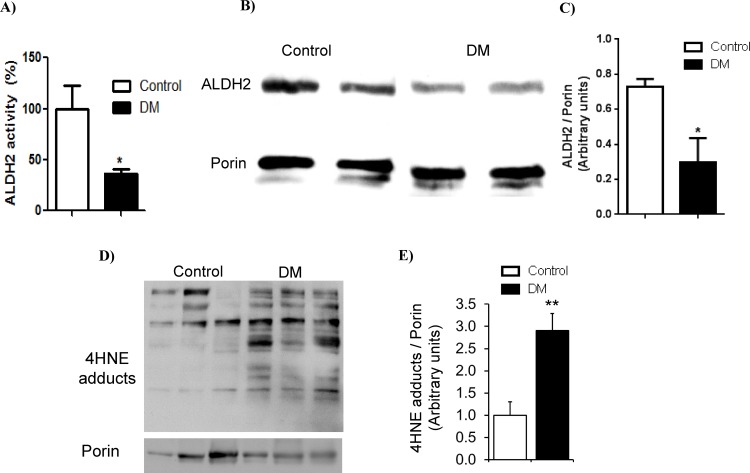
Change in myocardial ALDH2 levels, activity and 4HNE protein adducts. A. ALDH2 activity. The data expressed are mean ± SEM. N = 5–6 rats. * p<0.05 *vs*. Control. B. Western blot images of ALDH2 and porin. Porin, a mitochondrial protein, was used as a loading control to normalize mitochondrial ALDH2. C. The densitometry quantification data of ALDH2/porin. Protein lysates of cardiac homogenates were used for measuring ALDH2 levels by Western blotting in DM and control rats. The data expressed are mean ± SEM. N = 5–6 rats. * p<0.05 *vs*. Control. D. Western blot images of 4HNE adducts and porin. E. The densitometric quantification data of 4HNE protein adducts/porin. The data expressed are mean ± SEM. N>3 rats. * p<0.05 *vs*. Control.

Most importantly, myocardial ALDH2 activity (Fig 1A) and levels (Fig 1B and 1C) were reduced and 4HNE protein adduct levels (Fig 1D and 1E) were increased in diabetic hearts compared to control hearts.

### Reduction in mitochondrial respiration, their reserve capacity and levels of mitochondrial OXPHOS protein subunits of respiratory complex proteins in diabetic rat myocardium

**Fig 2:**
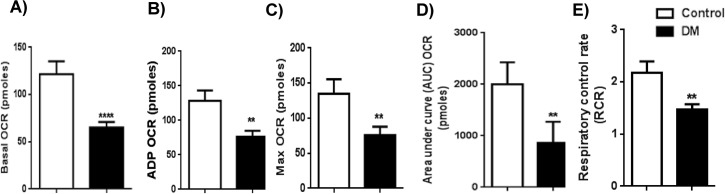
Mitochondrial respiration indices. Mitochondrial oxygen consumption rate (OCR) of control and DM rats: A) Mitochondrial basal OCR (Δ OCR from basal minus of Antimycin-A). B) ADP OCR (ADP minus Oligomycin). C) Maximum OCR (FCCP minus Antimycin-A). D) FCCP-induced mitochondrial respiratory reserve capacity. (Area under curve of FCCP minus area under curve oligomycin) was plotted as a graph. E) The ratio between state 3 and 4 respirations was depicted as the respiratory control rate (RCR). The data expressed are mean ± SEM. N = 6–11. ** *p* < 0.01 *vs* control;.***P<0.0001 *vs* control.

The mitochondrial oxygen consumption rate (OCR), an index of mitochondrial respiration, was decreased in mitochondria isolated from diabetic hearts compared to controls. We calculated mitochondrial basal OCR (Δ OCR from basal minus Antimycin-A; {122 ± 14 (control) *vs* 65 ± 6 (DM)} (Fig 2A), ADP OCR ADP minus Oligomycin; {128 ± 15 (control) vs 76 ± 8 (DM)} (Fig 2B), and maximum OCR FCCP minus Antimycin-A; {135 ± 21 (control) vs 76 ± 12 (DM)} (Fig 2C).

Most strikingly, the mitochondrial respiratory reserve capacity with FCCP (AUC OCR) was significantly decreased in the diabetic group with chronic hyperglycemia (860 ± 130) compared to the control group (2001 ± 429) (Fig 2D). Finally, the respiratory control rate (RCR) of state III/state IV was also significantly reduced in the diabetic group (1.5 ± 0.1) compared to the control group (2.2 ± 0.2) (Fig 2E). The RCR is a bit low even in the control groups, perhaps due to the old age of the rats (Fig 2E).

**Fig 3:**
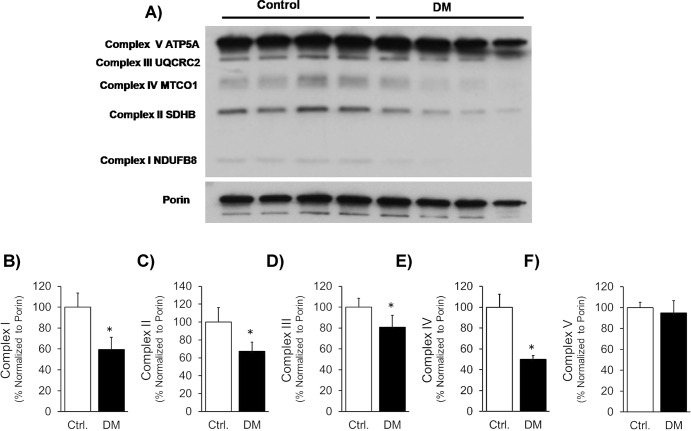
Levels of mitochondrial complex proteins. A). Western blot images of mitochondrial OXPHOS respiratory complex protein levels and VDAC (Porin) were shown in DM compared to control. A cocktail antibody comprising the following subunits of respiratory complex proteins are used: NADH dehydrogenase (ubiquinone) 1 beta subcomplex 8 (NDUFB8; complex I), succinate dehydrogenase complex, subunit B, iron sulfur (SDHB/Ip; complex II), ubiquinol-cytochrome c reductase core protein II (UQCR2; complex III), cytochrome c oxidase subunit 2 (COXII; complex IV) and ATP synthase 5A (ATP 5A, Complex V). B, C, D, E and F). Quantification of the levels of each of the above mentioned subunits were shown, respectively. The data was presented as % of proteins normalized to porin levels.

DM also reduced the levels of mitochondrial OXPHOS protein subunits of respiratory complex proteins (Fig 3A–3F). Specifically, ETC complex protein subunits NADH dehydrogenase (ubiquinone) 1 beta sub complex 8 (NDUFB8; complex I), succinate dehydrogenase complex, subunit B, iron sulfur (SDHB/Ip; complex II), ubiquinol-cytochrome c reductase core protein II (UQCR2; complex III), and cytochrome c oxidase subunit 2 (COXII; complex IV) were significantly reduced. ATP synthase 5A (ATP 5A, Complex V) was not decreased in DM.

### Chronic hyperglycemia increased pathological cardiac hypertrophy and fibrosis

**Fig 4:**
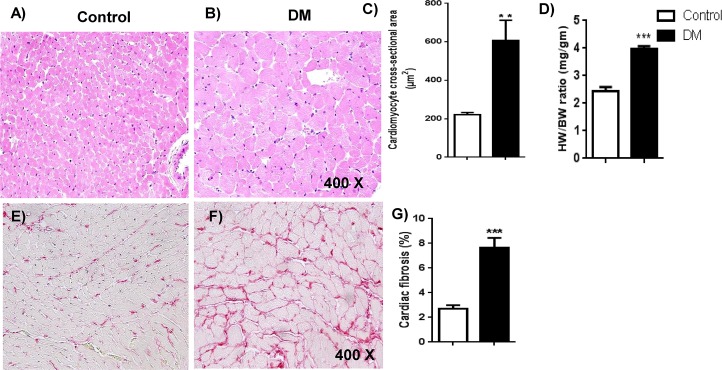
Histopathological analysis of myocardial hypertrophy and fibrosis. A & B: Cardiomyocyte hypertrophy: Photomicrographs of cardiac sections stained with hematoxylin-eosin from the control and DM groups were shown. The increase in cardiomyocyte size was apparent in the DM group. N = 5–6. C. The quantification data of cardiomyocyte cross-sectional area was shown. The data expressed are mean ± SEM. N = 5–6 **p <0.01. D. Quantification of heart weight to body weight ratio. E & F. Cardiac fibrosis. Representative micrographs of cardiac sections stained with Picrosirius red from the control and DM groups were shown. The red color area indicates collagen deposition in the heart. N = 5–6. G. Percent (%) area of cardiac fibrosis. The collagen deposition was quantified and presented as a % area of cardiac fibrosis. The data expressed are mean ± SEM. N = 5–6 ***p <0.0001.

There were increases in the cardiomyocyte cross sectional area (Fig 4A, 4B and 4C) and heart weight to body weight ratio (Fig 4D). Thus, cardiac hypertrophy was found in diabetic rats versus in control rats. Furthermore, increase in cardiac fibrosis was evident from increased collagen deposition (Fig 4E, 4F and 4G) with Picrosirius red in the diabetic cardiac sections compared to cardiac sections of the control group.

### Chronic diabetes reduced cardiac performance in streptozotocin-injected rats

The cardiac performance was reduced in diabetic rats compared to controls. As listed below in detail: 1) Fractional shortening was reduced to 35% in the diabetic condition from a control value of 45% (S1A and S1B Fig) 2) Left ventricular dimensions during systole and diastole (S1C and S1D Fig) were also increased in diabetic rats compared to control rats. 3) A 42% decrease in E/A ratio was recorded with the diabetic condition compared to the normal control (S2A and S2B Fig). 4) Left ventricular systolic pressure was reduced in the DM model compared to the control {97 ± 4 mmHg in diabetic group *versus* 129 ± 5% in the controls (S3A and S3B Fig)}. 5) Heart rate was lower in the diabetic heart (281 ± 9 beats per minute) compared to the control. (343.5 ± 12 beats per minute) (S3C Fig). 6) Left ventricular end diastolic pressure (LVEDP), a measure of diastolic function, was increased in the hyperglycemic condition (14.2 ± 1.1 mmHg) compared to the normal condition (8 ± 1.5 mmHg) (S3D Fig). 7) Peak and minimum LV dP/dt values were also reduced in the diabetic condition and this data was presented as +dP/dt and -dP/dt (S4A and S4B Fig).

### Reduced ALDH2 activity was correlated with mitochondrial dysfunction, pathological remodeling and cardiac dysfunction in rat diabetic myocardium

**Fig 5:**
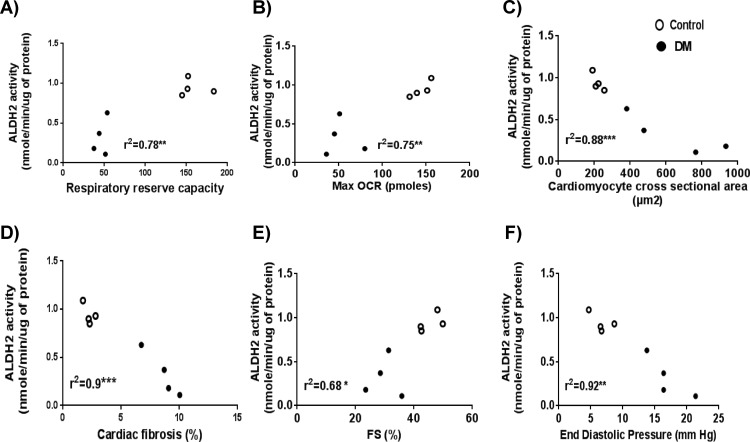
Correlation between ALDH2 activity and mitochondrial respiratory dysfunction, cardiac pathological remodeling and cardiac dysfunction. Graphs showing direct correlation between ALDH2 activity with A) Respiratory reserve capacity, B) Maximum OCR and inverse correlation with C) Hypertrophy, D) Fibrosis, E) Systolic dysfunction and F) diastolic dysfunction. Data were analyzed by linear regression (*** < 0.*001*, ** < 0.*01*, * p < 0.*05)*. N = 4 per group. Open and closed circles denote control and DM respectively.

Reduced ALDH2 activity was correlated with mitochondrial respiratory dysfunction {mitochondrial reserve capacity (Fig 5A) and maximal respiration (Fig 5B)}, pathological cardiac remodeling {cardiac hypertrophy (Fig 5C) and fibrosis (Fig 5D)} and cardiac dysfunction {%FS (Fig 5E) and end-diastolic pressure (Fig 5F)}.

## Discussion

In the present study, we found reduced ALDH2 activity correlated with decreased mitochondrial respiration and their reserve capacity in the myocardium of chronic hyperglycemic rats.

Molecular biochemical studies implicate oxidative stress as an important causative factor of diabetic complications [13, 28]. Hyperglycemia-induced ROS is reported to cause the generation of advanced glycated end products (26, 27) and reactive carbonyl compounds, including toxic aldehydes such as 4HNE (14, 15), malondialdehyde (MDA) (28) and methylglyoxal (MGO) (29). Several studies implicated reactive aldehydes in the pathophysiology of diabetes-induced tissue damage including heart (14), liver (30), kidney (31) and vasculature (32). In this study, we found increased 4HNE protein adducts in a diabetic rat myocardium. There was also reduced ALDH2 activity in the diabetic heart which may be due to 4-HNE adduct formation on ALDH2 as shown in a type-2 diabetic mouse model [15]. It has been shown that ALDH2 activity and levels were decreased in streptozotocin-induced diabetes in rats [21] and mice [20, 29]. However, no one has yet exclusively studied whether this reduction contributes to defective mitochondrial respiration.

ALDH2 has been implicated as a therapeutic target in cardiovascular diseases [30]. There are several studies using ALDH2 knockout mice that demonstrated the importance ALDH2 in cardio protection. For instance, at 4 weeks after MI, myocardial infarct size, cavity size and end-diastolic pressure were significantly greater and ejection fraction was significantly lower in ALDH2 knockout mice than in WT mice [31]. In a model of hind limb ischemia, the perfusion ratio was achieved 80% at the 3-week point in WT mice but only a 60% perfusion ratio was found in ALDH2 knockout mice [32]. In both these studies, transgenic restoration of ALDH2 ameliorated the pathologies. Patients lacking ALDH2 activity due to ALDH2*2 mutation show a strong association with coronary spastic angina [33]. All these studies demonstrate that ALDH2 is very critical in cardiovascular diseases. Pertaining to our model, type-1 DM induced diastolic dysfunction in ALDH2 knock out mice [34] while ALDH2 overexpression has been shown to attenuate type-1 DM-induced cardiac damage in mice [20]. Even though we demonstrate a significant correlation between ALDH2 inactivity with mitochondrial respiratory dysfunction, pathological cardiac remodeling and cardiac contractile dysfunction, future studies using genetic and pharmacological tools for ablation and activation of ALDH2 will strongly reaffirm the role of ALDH2 in diabetic cardiac damage and dysfunction.

The cardioprotective effect of ALDH2 came to the limelight after ALDH2 was identified as a target of protein kinase C (PKC) epsilon [19]; ALDH2 is phosphorylated by PKC epsilon and this phosphorylated ALDH2 has increased activity compared to non-phosphorylated ALDH2. There are specific phosphorylation sites identified in ALDH2 including Thr185, Thr412 and Ser 279 after interaction with PKC-epsilon [19]. Endogenous PKC-epsilon activation was showed to protect type-1 diabetic heart [35]. It was not determined whether ALDH2 phosphorylation was a causative factor for this effect. We anticipated a decrease in ALDH2 phosphorylation in our diabetic rat hearts. However, we did not find any decrease in ALDH2 phosphorylation in our samples as demonstrated by co-IP studies (S5 Fig). We speculate the phosphorylation event may be a temporal process and we could have missed it as we test it at a single time point (6 months of diabetes). A time course study may clarify the ALDH2 phosphorylation state in a time-dependent manner in chronic DM. Reduction in ALDH2 activity in the tissue can attenuate 4HNE metabolism, resulting in abnormally high levels of 4HNE accumulation and subsequent protein adduct formation. One of the important consequences of this effect is increased 4HNE adduct formation in critical mitochondrial proteins involved in mitochondrial respiration, [36] which may cause defective mitochondrial respiration.

As we elucidated in a review, aberrations in mitochondrial function and its regulatory process are critical in the development of heart failure/cardiomyopathy, including diabetes-induced cardiomyopathy/cardiac damage [37]. Mitochondrial dysfunction, such as uncoupling of the electron transport chain and oxidative phosphorylation, results in generation of cell-damaging ROS *in vitro* and *in vivo*. In this study, we evaluated mitochondrial respiration by measuring the OCR of isolated mitochondria from STZ-induced diabetic and control hearts. Specifically, we calculated mitochondrial respiratory reserve capacity as this was implicated as the index of oxidative stress-mediated mitochondrial dysfunction. When we found that there was a significant decrease in mitochondrial respiration in the diabetic condition, we suggested that the increased 4HNE and /or reduced ALDH2 activity should be responsible. In an earlier study by Hill *et al*. it was shown that 4HNE treatment in neonatal cardiomyocytes attenuated the mitochondrial respiratory reserve capacity [23]. This, however, is the first report to implicate reduced ALDH2 activity and impaired mitochondrial respiratory reserve capacity in an animal model of diabetic cardiomyopathy. Exhaustion of the mitochondrial reserve capacity will ultimately result in respiratory dysfunction in oxidative stress conditions. Therefore, our study point out a new important subcellular defect that occurs in the diabetic heart, along with ALDH2 impairment.

In the diabetic heart, hyperglycemia-induced 4HNE adduct formation on ALDH2 can reduce its activity. In turn, the reduced ALDH2 activity will lead to lowered 4HNE detoxification. Thus a vicious cycle sets in, ultimately resulting in decreased mitochondrial respiration, presumably by forming adducts with critical mitochondrial complex proteins. Earlier studies demonstrated that 4HNE specifically forms adducts with mitochondrial proteins such as α-ketoglutarate dehydrogenase [38, 39], and inhibits NADH-linked respiration by reducing the steady-state level of NADH in isolated cardiac mitochondria [39]. We have summarized such findings in a recent review [14]. The oxidative phosphorylation, a key step in ATP generation in mitochondria is carried out by a set of protein complexes in the electron transport chain. More precisely, 4-HNE has been shown to form adducts with mitochondrial complex proteins themselves like with succinyl dehydrogenase (SDH) in the diabetic heart [22] and cytochrome c oxidase in the ischemic heart [16]. Similarly, in the current study, we noticed a significant reduction in 4 mitochondrial respiratory complex proteins in diabetic hearts to varying extents. These specific respiratory complex proteins are NADH dehydrogenase (ubiquinone) 1 beta subcomplex 8 (NDUFB8; complex I), succinate dehydrogenase complex, subunit B, iron sulfur (SDHB/Ip; complex II), ubiquinol-cytochrome c reductase core protein II (UQCR2; complex III) and cytochrome c oxidase subunit 2 (COXII; complex IV). The reduction in these ETC complex proteins in diabetes may be due to 4HNE adduct formation on them. This reduction in the ETC complex proteins could be the reason for the lower mitochondrial respiration that we observed in the diabetic heart.

In a model of STZ-induced DM, occurrence of diastolic dysfunction was due to mitochondrial abnormalities such as reductions in ATP synthesis, mitochondrial Ca^2+^ uptake and state-3 respiration [40]. Mitochondrial dysfunction [41, 42] was shown to contribute to myocardial contractile abnormalities. These studies implicate that cardiac dysfunction in the myocardium can be due to mitochondrial malfunction. Our diabetic rats exhibited both diastolic and systolic dysfunction which should be partly due to mitochondrial respiratory malfunction that we observed in those hearts. Further, we found reduced ALDH2 activity in the diabetic heart that may have been linked with the poor mitochondrial respiration and cardiac contractile dysfunction.

We also observed cardiac hypertrophy and fibrosis with the chronic hyperglycemia in rat hearts. Cardiac hypertrophy in diabetic myocardium may be due to increased cardiomyocyte death. Cardiac fibrosis is another important remodeling event in the diabetic heart [43] which was demonstrated to be enhanced by 4HNE [44, 45] and AGEs [46]. Fibrosis and hypertrophy in the diabetic heart are major events in the pathological ventricular structural remodeling, and will ultimately lead to diastolic and systolic dysfunction. In turn, poor contractility can fuel progressive pathological remodeling and heart failure. Even though there are multiple stress signals, signaling pathways and subcellular targets are proposed for these processes, ALDH2 inactivity has been correlated with pathological remodeling and poor cardiac function by us [15] and others [21] earlier as well as in the current study. Our correlation graphs indicate the inverse relationship between ALDH2 activity and mitochondrial dysfunction and also with pathological remodeling and cardiac dysfunction. Such correlation analysis point out future directions. We believe that our study suggests chronic hyperglycemia-induced 4HNE toxicity imparts functional changes at the organelle, cell and organ levels in the myocardium. Therefore, removal of 4HNE may be a potential strategy to mitigate hyperglycemia-induced cardiac damage and dysfunction from the molecular and cellular level.

Finally, we want to conclude that chronic hyperglycemia-induced impaired ALDH2 activity is associated *at least* partially for the decreased mitochondrial respiration and ultimately enhanced hypertrophy, fibrosis, and cardiac systolic and diastolic dysfunction in the diabetic myocardium.

## Supporting Information

S1 Fig Echocardiographic data. A. M-mode echocardiographic images. Representative echocardiographic images of control and DM groups were shown. IVS-intraventricular septum; LVDs-Left ventricular dimension during systole; LVDd-Left ventricular dimension during diastole. B. Fractional shortening. The data expressed are mean ± SEM. N = 6 ***p <0.0001. C. Left ventricular dimension during systole. The data expressed are mean ± SEM. N = 6 ***p <0.0001. D. Left ventricular dimension during diastole. The data expressed are mean ± SEM. N = 6 **p <0.0001
(TIFF)

S2 Fig Trans mitral filling velocities by tissue Doppler. A. Doppler echocardiographic images. Representative Doppler echocardiographic images of the control and DM groups. E, early and A, late waves were identified in the image depicting diastolic filling velocities. B. Quantification data of E/A ratio. In the ratio between early (E) and late (A) diastolic filling velocities, the E/A ratio was plotted as a graph. The data expressed are mean ± SEM. N = 6 ***p <0.0001
(TIFF)

S3 Fig Hemodynamic measurements. A. Tracing of a lab chart recording of the left ventricular systolic pressure. A representative tracing from control and DM rats was shown. B. The quantification data of the left ventricular systolic pressure. The data expressed are mean ± SEM. N = 6 ***p <0.0001. C. Heart rate. The data expressed are mean ± SEM. N = 6 ***p <0.0001. D. The quantification data of the left ventricular end diastolic pressure. The data expressed are mean ± SEM. N = 6 ***p <0.0001.
(TIFF)

S4 Fig Left ventricular systolic pressure peak and minimum (+dP/dt and -dP/dt). A. Tracing of lab chart recording of the left ventricular systolic pressure peak (+dP/dt) and minimum (-dP/dt). Representative tracings for +dP/dt and -dP/dt from control and DM rats was shown. B. The quantification data of the left ventricular systolic pressure peak (+dP/dt) and minimum (-dP/dt) from control and DM rats. The data expressed are mean ± SEM. N = 6 ***p <0.0001
(TIFF)

S5 Fig Immunoprecipitation of anti-Pser/Thr (phe) with ALDH2. The immunoblot image show representative control and diabetic samples along with beads with antibody and input (+ve control).
(TIFF)
