# Supplementary figures and images for: Cardiac Mitochondrial Respiratory Dysfunction and Tissue Damage in Chronic Hyperglycemia Correlate with Reduced Aldehyde Dehydrogenase-2 Activity
Source: PLoS One. 2016 Oct 13;11(10):e0163158. doi: 10.1371/journal.pone.0163158 (PMC5063328; doi:10.1371/journal.pone.0163158)

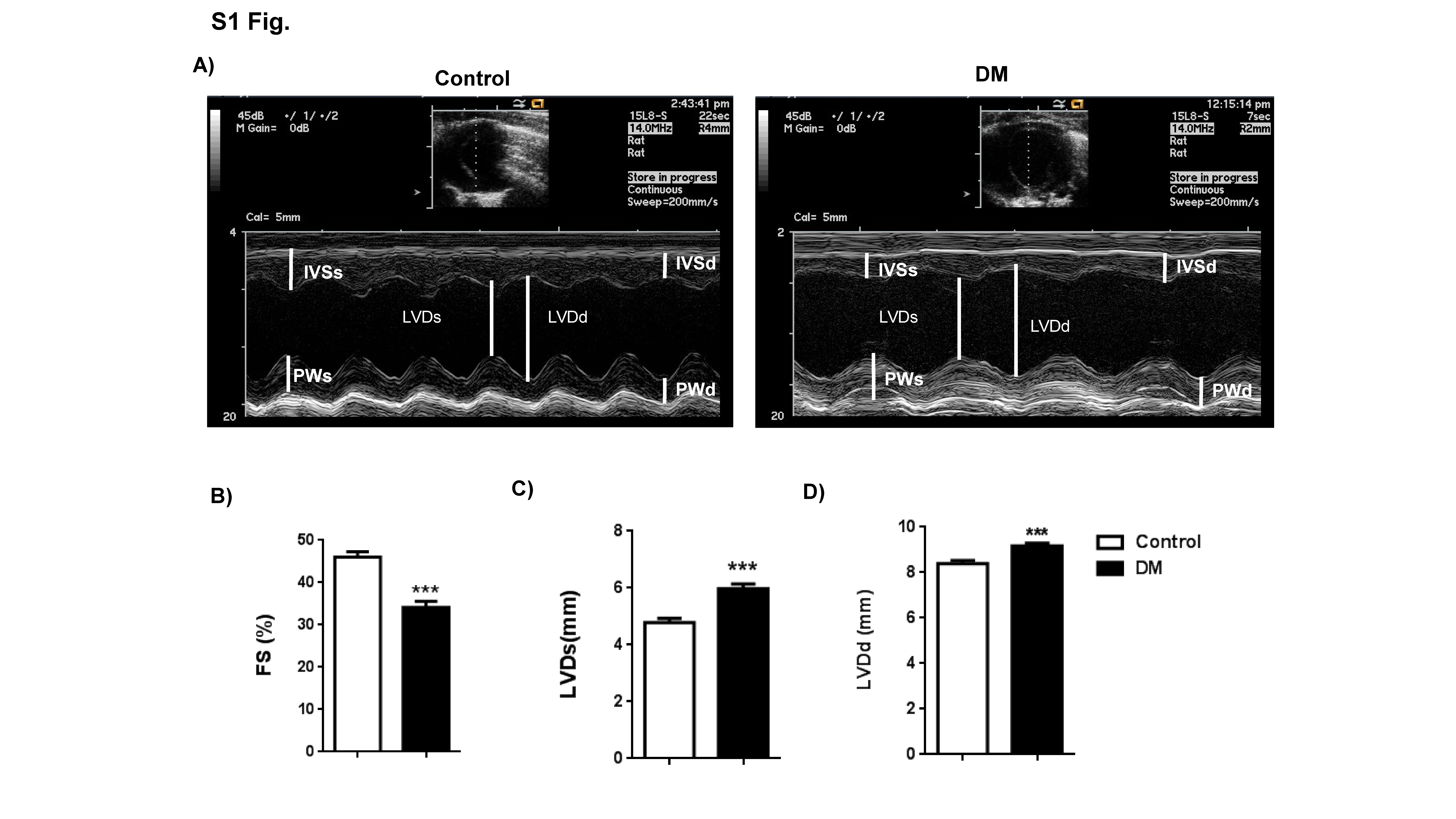

Supplement: S1 Fig — A. M-mode echocardiographic images. Representative echocardiographic images of control and DM groups were shown. IVS-intraventricular septum; LVDs-Left ventricular dimension during systole; LVDd-Left ventricular dimension during diastole. B. Fractional shortening. The data expressed are mean ± SEM. N = 6 ***p <0.0001. C. Left ventricular dimension during systole. The data expressed are mean ± SEM. N = 6 ***p <0.0001. D. Left ventricular dimension during diastole. The data expressed are mean ± SEM. N = 6 **p <0.0001 (TIFF) [file pone.0163158.s001.tiff]

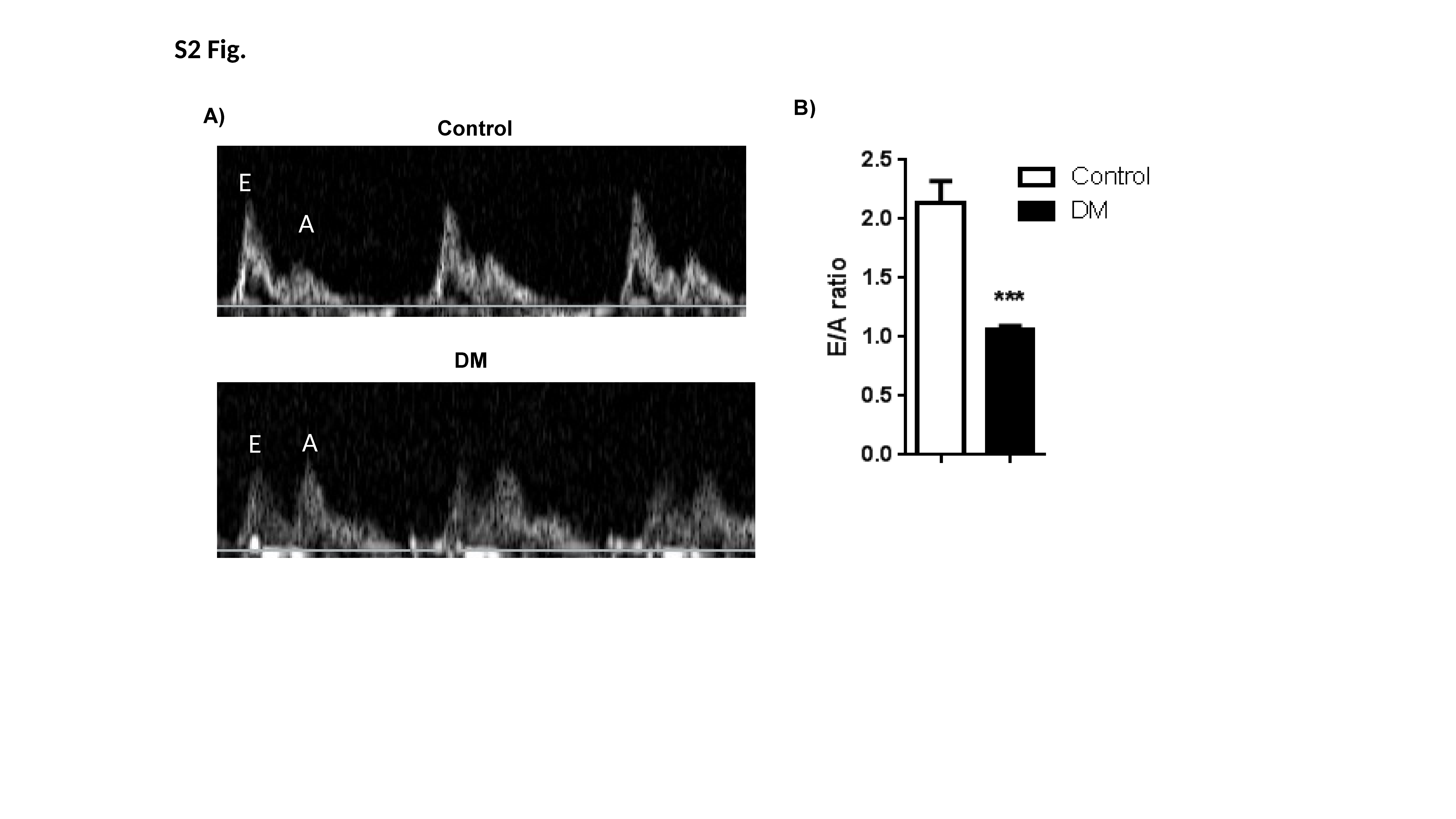

Supplement: S2 Fig — A. Doppler echocardiographic images. Representative Doppler echocardiographic images of the control and DM groups. E, early and A, late waves were identified in the image depicting diastolic filling velocities. B. Quantification data of E/A ratio. In the ratio between early (E) and late (A) diastolic filling velocities, the E/A ratio was plotted as a graph. The data expressed are mean ± SEM. N = 6 ***p <0.0001 (TIFF) [file pone.0163158.s002.tiff]

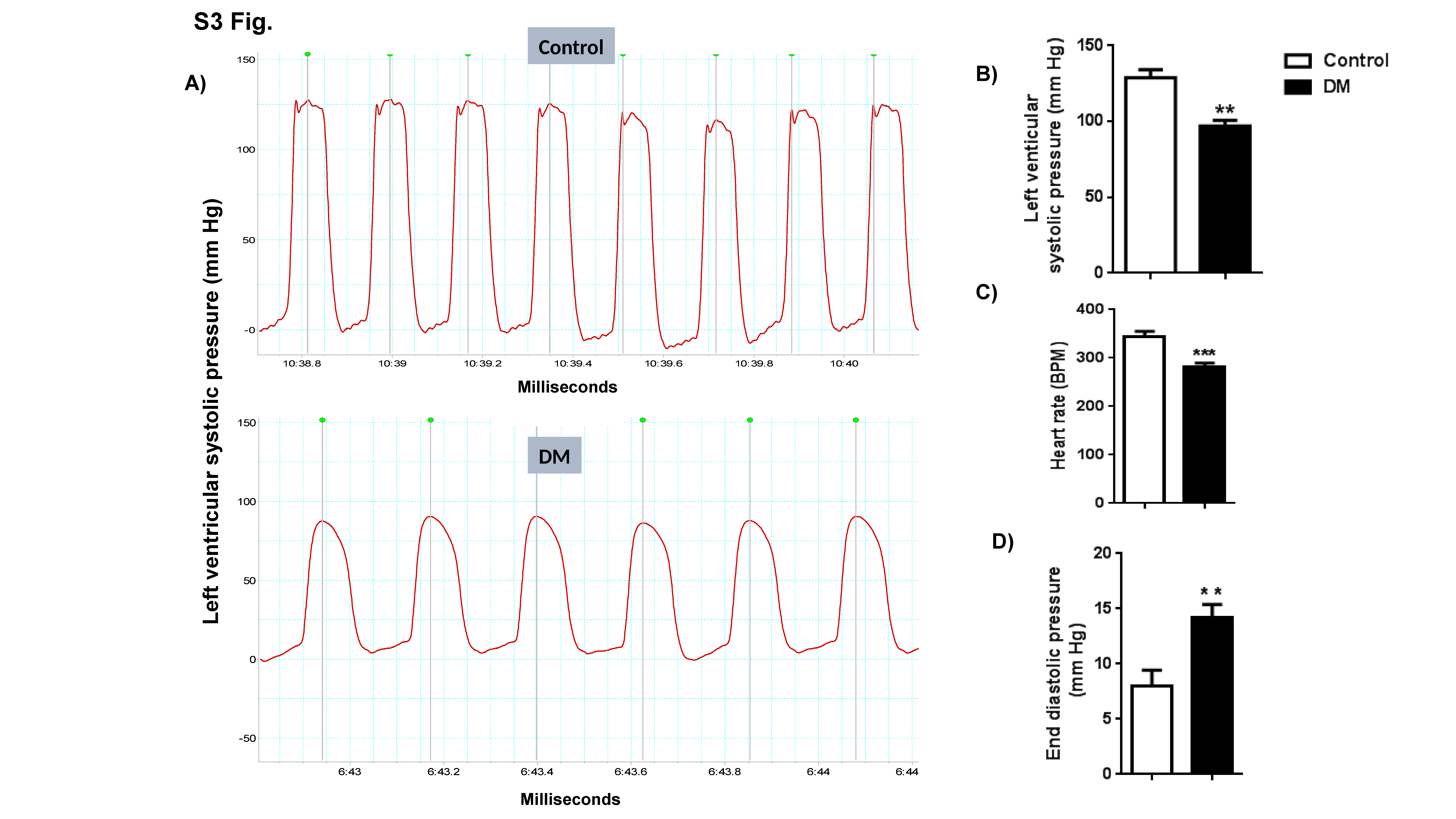

Supplement: S3 Fig — A. Tracing of a lab chart recording of the left ventricular systolic pressure. A representative tracing from control and DM rats was shown. B. The quantification data of the left ventricular systolic pressure. The data expressed are mean ± SEM. N = 6 ***p <0.0001. C. Heart rate. The data expressed are mean ± SEM. N = 6 ***p <0.0001. D. The quantification data of the left ventricular end diastolic pressure. The data expressed are mean ± SEM. N = 6 ***p <0.0001. (TIFF) [file pone.0163158.s003.tiff]

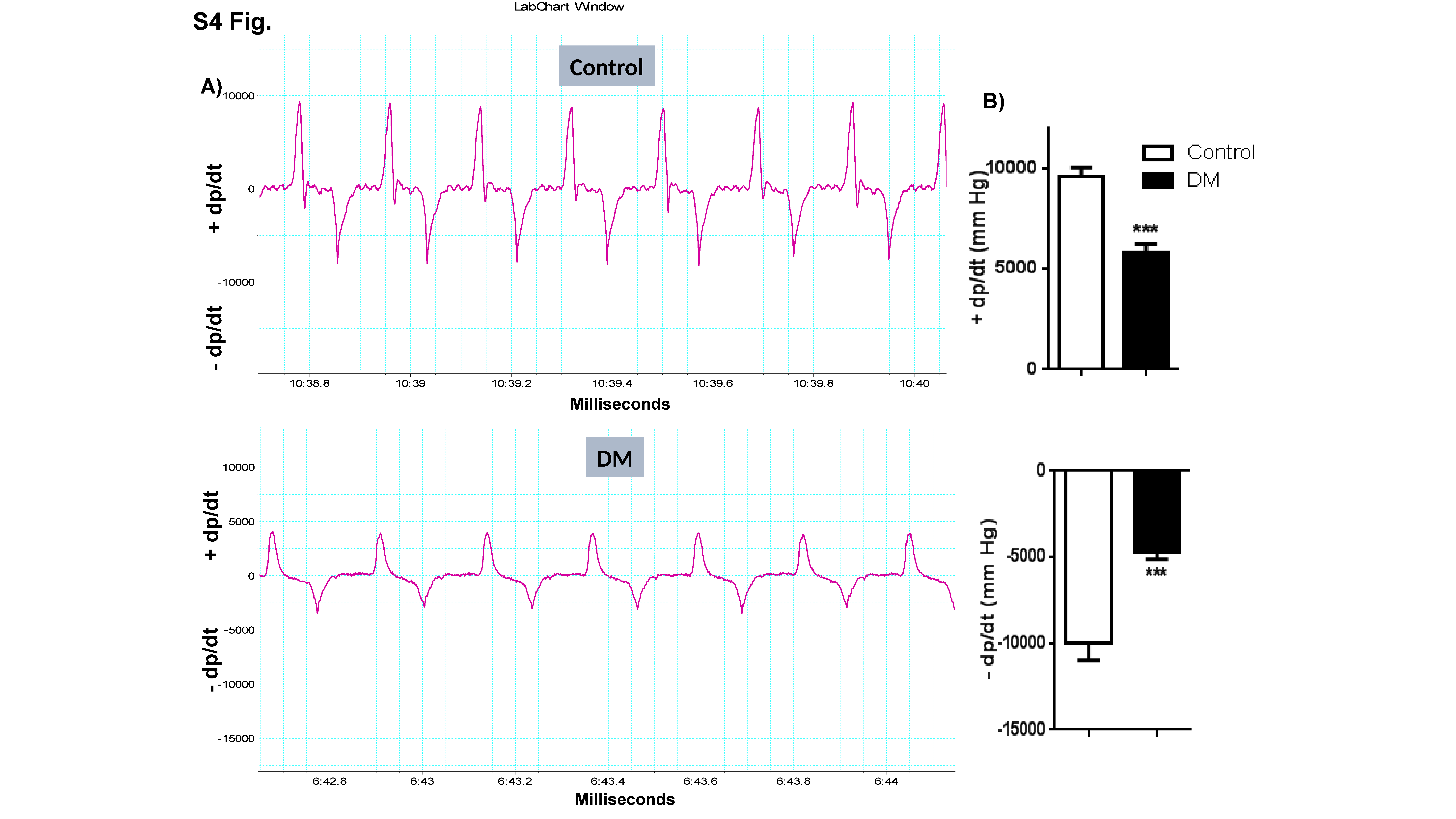

Supplement: S4 Fig — A. Tracing of lab chart recording of the left ventricular systolic pressure peak (+dP/dt) and minimum (-dP/dt). Representative tracings for +dP/dt and -dP/dt from control and DM rats was shown. B. The quantification data of the left ventricular systolic pressure peak (+dP/dt) and minimum (-dP/dt) from control and DM rats. The data expressed are mean ± SEM. N = 6 ***p <0.0001 (TIFF) [file pone.0163158.s004.tiff]

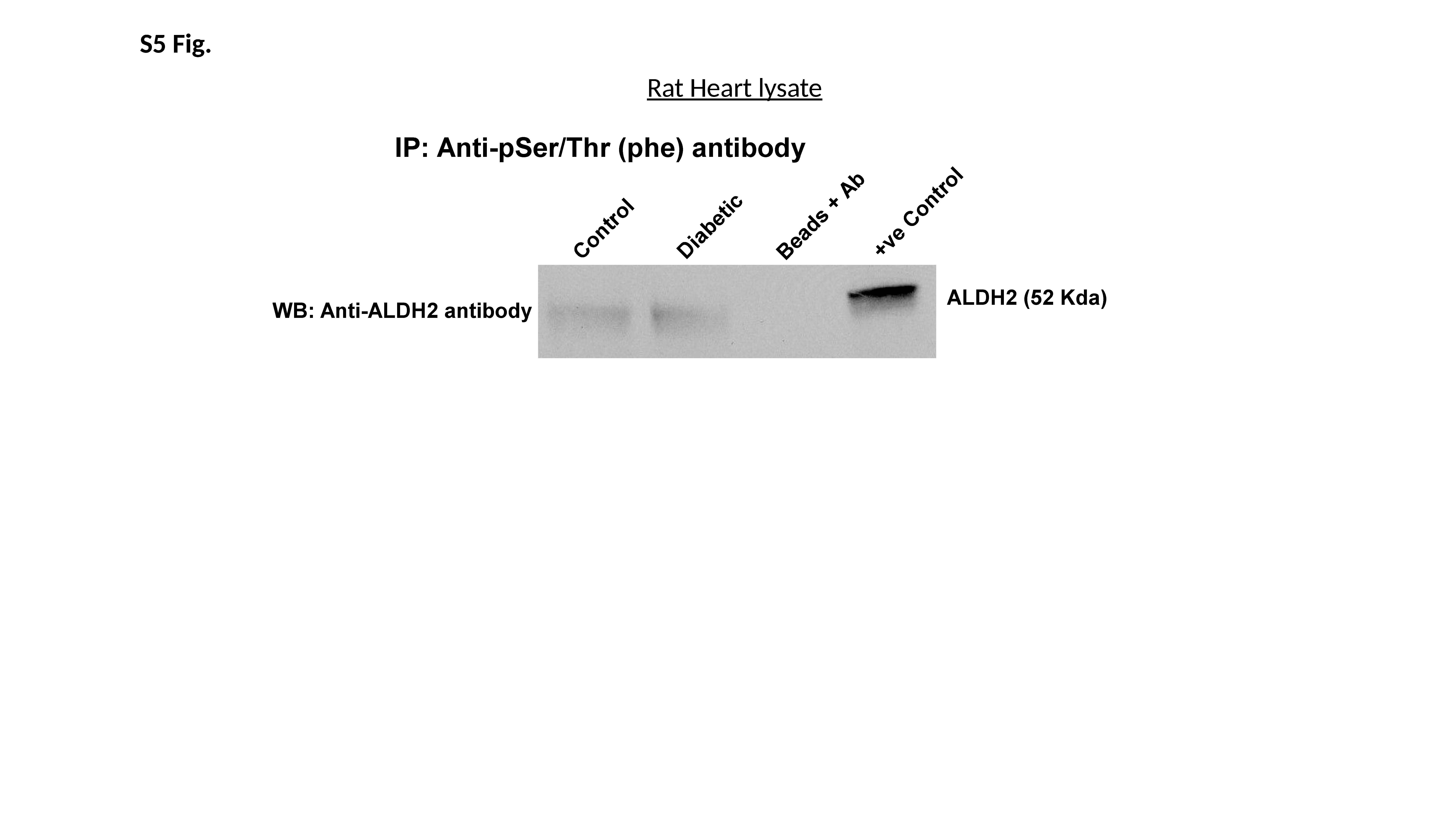

Supplement: S5 Fig — The immunoblot image show representative control and diabetic samples along with beads with antibody and input (+ve control). (TIFF) [file pone.0163158.s005.tiff]
